# Allen and Hanburys' Bicentenary

**Published:** 1916-01-22

**Authors:** 


### ALLEN AND HANBURYS' BICENTENARY.

The firm of Allen and Hanburys, Limited, was established in the City of London in the year 1715, and in order to celebrate the bicentenary a meeting of the employees was recently held at Liverpool Street Hotel, London, when a presentation was made to the vice-chairman of the company—Mr. Frederick Janson Hanbury—of his portrait in oils by Mr. Percy Bigland, and to a co-director, Mr. W. Ralph Dodd, of a cabinet of silver, Mr. F. W. Gamble, another director, in making the presentations, referred to some of the early associations of the firm, mentioning that the foundations of the Plough Court pharmacy—where the business, which is now world-famous, had its origin—were laid by Silvanus Bevan. In later years the business was conducted by William Allen, F.R.S., the first President of the Pharmaceutical Society, and it is interesting to note that in his day the Plough Court pharmacy was the meeting-place of Lord Brougham, Wilberforce, Clarkson, and other pioneers of the anti-slavery movement. Another eminent man associated with the firm was Daniel Hanbury, F.R.S., whose name and work are commemorated by the Hanbury Medal, awarded every two years to the most distinguished international worker in pharmacognosy, Daniel Hanbury's favourite subject.

